# Laparoscopic Management of Isolated Splenic Hydatid Disease in a Rural Centre: A Case Report

**DOI:** 10.7759/cureus.34206

**Published:** 2023-01-25

**Authors:** Stephanie E Cornish

**Affiliations:** 1 General Surgery, University of Queensland, Brisbane, AUS; 2 General Surgery, Toowoomba Base Hospital, Toowoomba, AUS

**Keywords:** spleen, laparoscopic, rural, hydatid disease, splenic hydatidosis

## Abstract

Hydatid disease affects a significant proportion of the world’s population, particularly in endemic areas, with substantial associated morbidity and mortality. However, the incidence of splenic involvement is rare, and isolated splenic disease is even more so; it is infrequently encountered in western countries. As such, their management is often limited to metropolitan tertiary centres with no fixed consensus on the best surgical approach. The author presents a case of the successful management of isolated splenic hydatid disease using a laparoscopic approach in a rural facility, in what is believed to be the first presented Australian case of isolated splenic hydatidosis.

## Introduction

Hydatidosis is a chronic zoonotic disease that has a significant impact on public health due to its substantial morbidity and mortality, and subsequent economic ramifications for affected communities, to the point where the World Health Organisation listed it in the 2008-2015 strategic plan for control of Neglected Tropical Diseases (NTDs) [1]. It is endemic in areas where husbandry is common but access to veterinary services is poor, including parts of South America, the Mediterranean, the Middle East, and Africa, with a worldwide annual incidence of 1-200 per 100,000 [2]. In Australia, the incidence is much lower, at only 0.2 cases per 100,000 [3]. Hydatidosis is a parasitic infection caused by the cystic stage of the small taeniid-type tapeworm Echinococcus (E.), more often E. granulosus than E. multilocularis, wherein humans are an intermediate host infected through the incidental ingestion of the parasite’s eggs excreted in dog faeces via hand-to-mouth transfer [1-2,4]. Hydatid disease can affect any part of the human body, and most frequently affects the liver (60-70%) and the lungs (20-30%), while splenic hydatid disease represents only 0.5-4% of cases, despite being the third most commonly affected organ; isolated splenic hydatid cysts are even rarer [5].

## Case presentation

**Figure 1:**
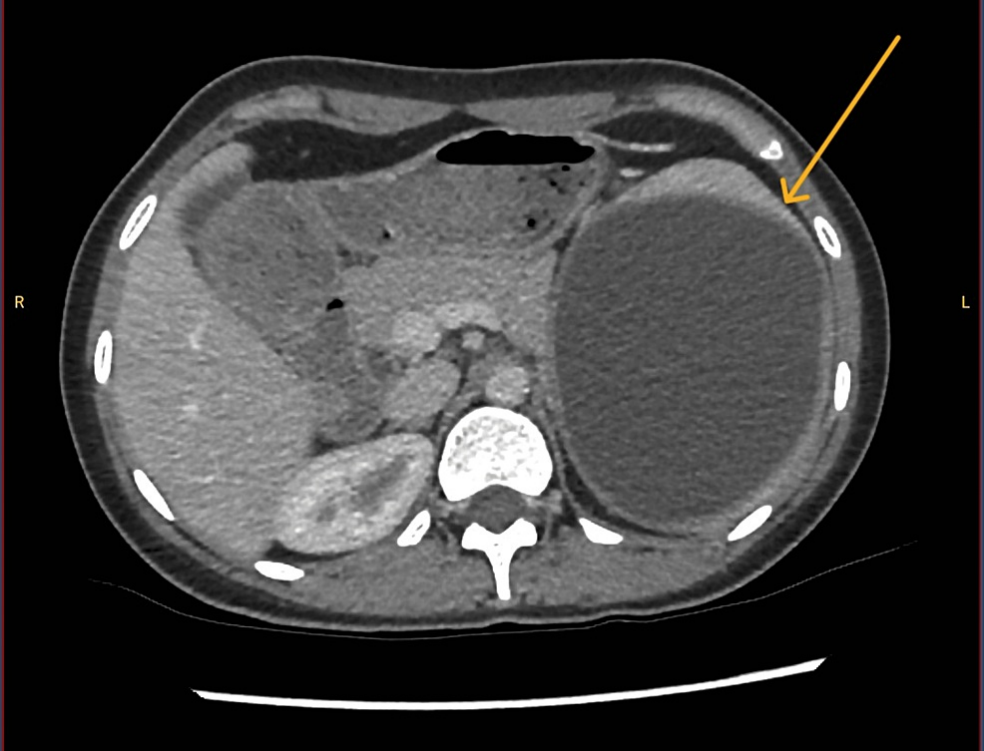
Axial view of CT portal venous showing the size of the isolated splenic hydatid cyst and associated mass effect Arrow indicates a small proportion of the remaining splenic parenchyma, encasing a large cyst

A 29-year-old female was seen in the general surgery outpatient clinic of a rural hospital for six years of left upper quadrant pain, but no other associated symptoms. This was in the context of previous exposure to sheep and dogs prior to immigrating from Iraq. She was otherwise well, with no comorbidities and no history of trauma or previous abdominal surgery. Examination revealed a palpable spleen, with tenderness in the left upper quadrant and flank. Preliminary investigation in the community included positive Strongyloides immunoglobulin G, and as such, arrangements were made for further investigation, including hydatid serology (positive), abdominal ultrasound (non-specific, left, upper quadrant cystic changes) and CT portal venous of the abdomen and pelvis, which showed a 150x180x100 mm well-defined cyst with homogenous fluid density (Figure 1). The patient was accordingly referred to the facility’s infectious diseases clinic for sterilisation of the cyst and thence commenced on albendazole.

**Figure 2:**
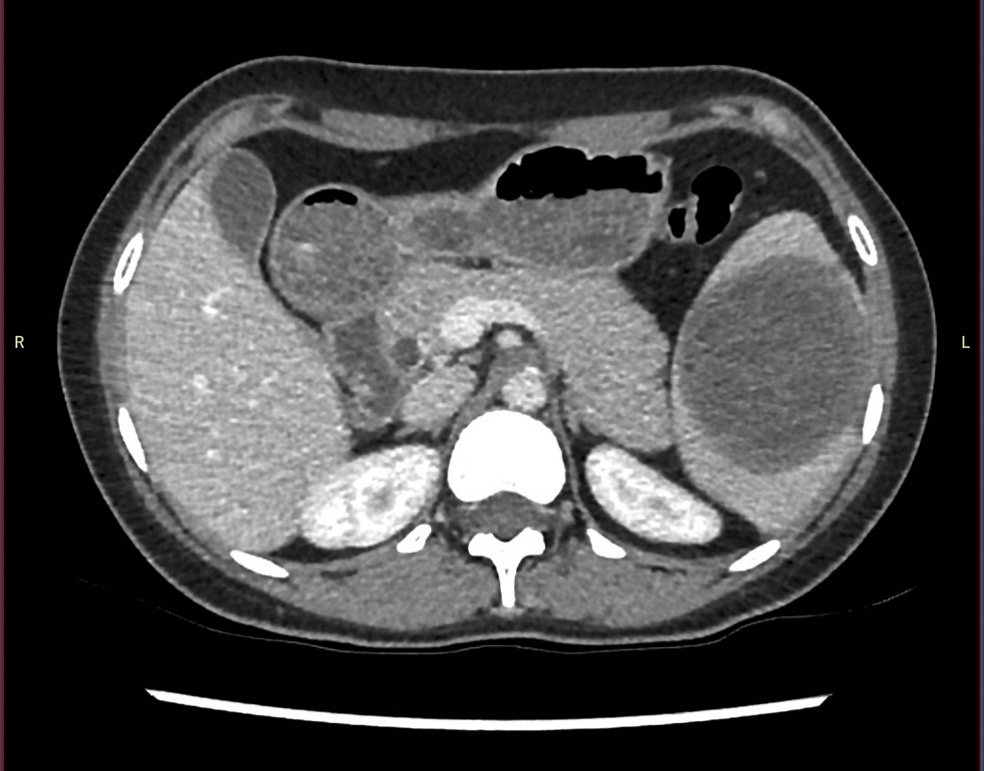
Serial CT imaging showing an interval decrease in the size of the cyst due to pharmacological interventions

Despite counselling regarding the associated risks, the patient subsequently became pregnant, requiring the cessation of albendazole. An acute admission for progressive left abdominal pain was then required, and the risks of cyst rupture in pregnancy were reinforced; however, the patient elected to continue with the pregnancy and was discharged following adequate analgesia. Following the successful completion of her pregnancy, a planned surgical outpatient review was performed, and the patient was found to be experiencing ongoing symptoms with the addition of daily subjective fevers, and as such was recommenced on albendazole. This was well-tolerated; however, after a continuous six-month course of treatment, she required further admission for pain. At this time, repeat cross-sectional imaging (Figure 2) showed an interval reduction in the size of the cyst to 79x63x77 mm; however, given the increase in symptomatology, a discussion was held between the treating surgical and infectious disease teams and with the district’s quaternary hepatobiliary service, and a consensus was reached to proceed to splenectomy.

**Figure 3:**
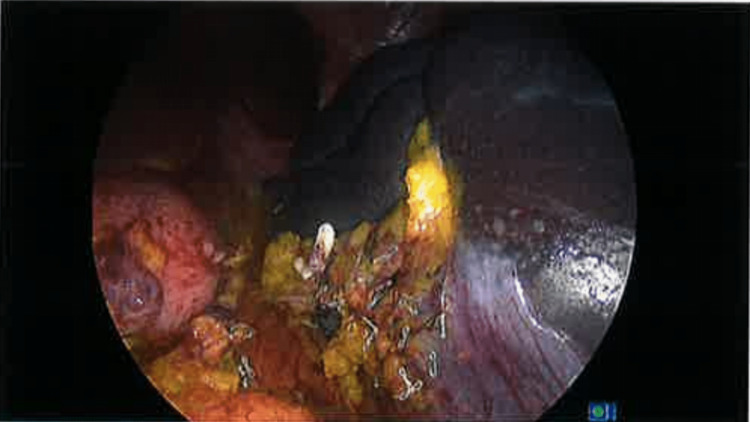
Intraoperative laparoscopic photograph showing considerable splenomegaly A Hemoloc clip and Endo-GIA linear staples are visible post-control and dissection of the splenic artery and vein.

**Figure 4:**
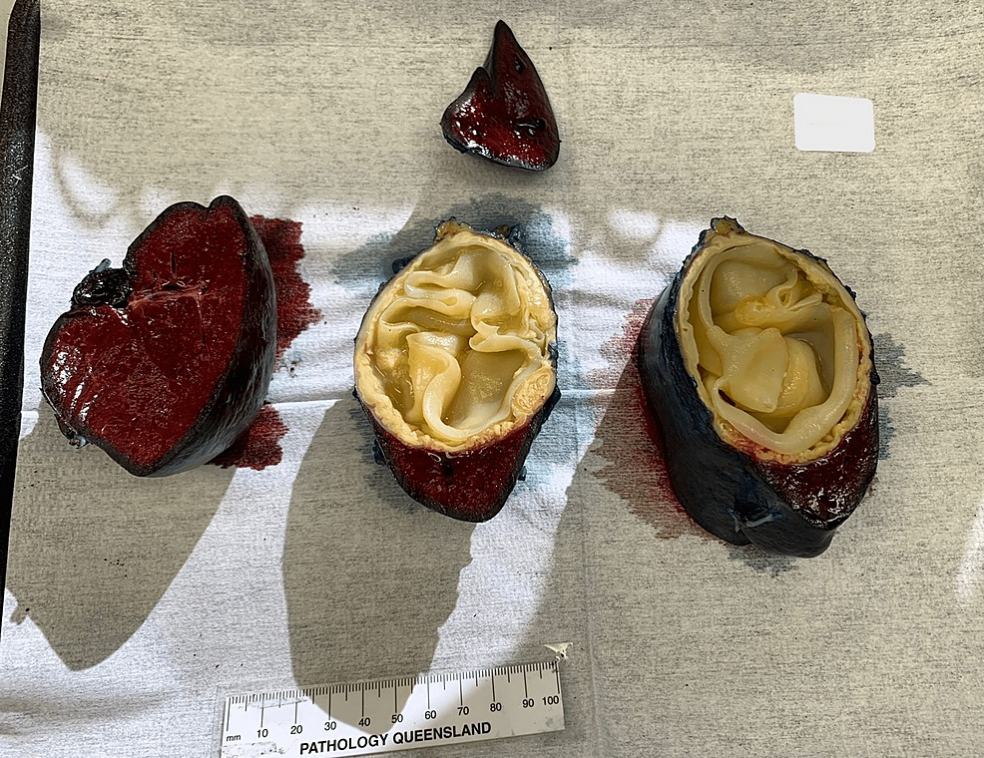
Cyst containing the spleen, successfully resected and removed The image shows the spleen once received at the facility's pathology lab, cut into four in preparation for sectioning and analysis.

A laparoscopic-assisted splenectomy was performed later that month using a standard 10 mm Hassan entry and four other ports were placed under vision. The gastrosplenic ligament was taken down, and the splenic artery and vein were identified and controlled using a Hemoloc clip to the artery followed by an Endo-GIA 60 mm linear stapler across both the artery and vein (Figure 3). Inferior dissection of the splenocolic ligament and subsequent medial-to-lateral dissection of the spleen with the division of the splenophrenic ligament allowed for the liberation of the spleen, and the spleen delivered whole in an Endocatch bag via a Pfannenstiel incision with no rupture or spillage and no immediate postoperative complications (Figure 4). Appropriate vaccinations as per Spleen Australia guidelines had been performed in preparation. The patient was discharged on postoperative Day 5. Routine cross-sectional imaging was performed six weeks later, which showed no evidence of further hydatid disease but did reveal a portal vein thrombus extending into the right lobe of the liver. She was subsequently admitted to facilitate a prompt hematological review and was commenced on three months of oral anticoagulation with complete resolution of the thrombus on subsequent imaging. An ongoing outpatient review has been performed, with discharge from the infectious disease service due to completed management of her hydatid disease, and further surgical review due at the end of the year.

## Discussion

Hydatid cysts of the spleen are usually slow growing and may be asymptomatic for long periods of time, with the onset of symptoms corresponding with the eventual impingement of surrounding organs, or development of complications such as elevated pressure within the cyst leading to rupture, secondary infection, intracystic haemorrhage, or fistulisation of the cyst [5]. Symptoms include equivocal discomfort and fullness of the upper abdomen, early satiety, dyspepsia, a palpable mass, pleuritic chest pain, shortness of breath, and impingement symptoms of the kidney, although non-specific pain is by far the most common [5]. Conversely, a significant proportion of cases are detected incidentally due to their insidious and slow growth [5]. The exact mechanism of splenic involvement is not known, however, theories include the escape of eggs before entering the systemic circulation, retrograde spread via the portal and splenic veins, and colonic trans-parietal passage of eggs or intraperitoneal rupture of an existing hepatic hydatid cyst [4].

Treatment of splenic hydatid cysts includes conservative, percutaneous, and surgical options [4]. Small, asymptomatic cysts may be managed with anti-helminthic drugs alone, such as albendazole, however, they require close follow-up [5]. Percutaneous drainage usually follows sterilisation with said anti-helminthic agents [5]. Furthermore, anti-parasitic agents are also required pre- and postoperatively in cases of surgical management in order to reduce rates of relapse [1].

Surgical management of splenic hydatidosis tends to be preferred for symptomatic or larger lesions due to the risk of spontaneous or traumatic rupture and is divided between total splenectomy and organ-preserving procedures; however, the limited available literature varies between which is the superior approach, with some arguing the superior eradication rates and reduced risk of rupture in total splenectomy and others advocating for spleen-sparing measures due to the rates of sepsis-related mortality post-splenectomy [4-5]. Spleen-sparing techniques tend to be used for smaller, peripheral cysts and include partial splenectomy, enucleation, deroofing with omentoplasty, internal drainage with cystojejunostomy, or external drainage [5]. In the case put forward, the isolated cyst encountered was peripheral in its location in the lower pole, however, its size and associated risk of rupture, along with ready access to prophylactic vaccinations and sepsis management, should it be required, led to a consensus for total splenectomy over possible organ-sparing techniques.

The next area of discussion is the use of open versus the less invasive techniques of laparoscopic and robotic approaches. In more established facilities, laparoscopic approaches and the technical expertise required are commonplace and often selected, as they are considered to be a superior technique that is minimally invasive with a quicker recovery whilst maintaining safe and effective removal of hydatid cysts [5]. However, the literature suggests that in developing countries in which the required expertise for safe laparoscopic techniques is less frequent, hesitancy and reluctance remain due to fear of rupture leading to intraperitoneal dissemination of cystic contents and resultant anaphylactic shock and recurrence of hydatidosis, thus a preference for open approaches in these situations [5]. Whilst not a quaternary hepatobiliary service, the treating facility, in this case, had the adequate capacity and technical ability to safely perform a laparoscopic approach with externalisation of the cyst-containing spleen through a Pfannenstiel incision, thus illustrating the potential for safe and effective management of isolated splenic hydatid disease in non-metropolitan areas so long as the treating surgeon possesses the necessary experience and expertise.

## Conclusions

The incidence and associated management of isolated splenic hydatid disease is rare, even more so in non-endemic areas. However, given appropriate expertise and access to facilities, it can be safely and effectively managed laparoscopically in a non-tertiary centre, given the involvement of the multi-disciplinary team and the local hepatobiliary service, with no detriment to safety or outcome.
